# The Japanese version of the Richards‐Campbell Sleep Questionnaire: Reliability and validity assessment

**DOI:** 10.1002/nop2.252

**Published:** 2019-03-28

**Authors:** Hiroaki Murata, Yoko Oono, Masamitsu Sanui, Keita Saito, Yoko Yamaguchi, Masanori Takinami, Kathy C. Richards, Richard Henker

**Affiliations:** ^1^ School of Nursing at Narita International University of Health and Welfare Chiba Japan; ^2^ Department of Intensive Care Unit Jikei University Hospital Tokyo Japan; ^3^ Department of Anesthesiology and Critical Care Medicine Jichi Medical University Saitama Medical Center Saitama Japan; ^4^ School of Nursing University of Texas at Austin Austin Texas; ^5^ Department of Nurse Anesthesia, School of Nursing University of Pittsburgh Pittsburgh Pennsylvania

**Keywords:** intensive care unit, Japan, polysomnography, Richards‐Campbell Sleep Questionnaire, sleep

## Abstract

**Aims:**

The purpose of this study was to determine the reliability and validity of the Japanese version of the Richards‐Campbell Sleep Questionnaire as a measure of sleep among intensive care unit patients in a Japanese hospital.

**Design:**

Cross‐sectional survey.

**Methods:**

The Richards‐Campbell Sleep Questionnaire was initially translated into Japanese using the back‐translation method. Validity was evaluated by determining the association between sleep efficiency, measured using simplified polysomnography, and the total score on the Japanese version of the Richards‐Campbell Sleep Questionnaire. Adult non‐intubated intensive care unit patients who completed the five‐item visual analogue scale underwent polysomnography for one night. Reliability was tested using Cronbach's alpha coefficient.

**Results:**

Thirty‐three patients were included in the analysis. After excluding four patients with subsyndromal delirium, the Pearson correlation coefficient was 0.602 (*p* = 0.001). Cronbach's alpha coefficient was 0.911.

**Conclusion:**

The Japanese version of the Richards‐Campbell Sleep Questionnaire could be used as an alternative to polysomnography when assessing sleep quality in lucid intensive care unit patients.

## 1 INTRODUCTION

The quality of sleep is impaired in critically ill patients (Friese, 2008; Murata & Yamaguchi, 2018; Richards, O'Sullivan, & Phillips, 2000). Sleep disorders can potentially cause hypersensitivity to pain, decline in immune system function, decreased cognitive function, increased mortality, increased length of stay in the hospital (Friese, 2008), conversion from non‐invasive positive‐pressure ventilation to intubation (Murata, Inoue, & Takahashi, 2017) and irregular endocrine function (Matthews, 2011). The sleep disorders cannot be ignored by nurses of intensive care unit (ICU).

The gold standard for the evaluation of sleep quality has been polysomnography (PSG); however, this requires long monitoring hours, bears a high cost and requires cooperation from patients. The other objective measurements of sleep in the ICU are done by using actigraphy. Actigraphy is a device that is similar to a wristwatch and less invasive device than PSG. Although actigraphy does not provide the accuracy of sleep stages, this is provided by PSG (van der Kooi et al., 2013).

As an alternative measure of sleep quality in the ICU, questionnaires such as the Verran–Snyder‐Halpern Sleep Scale (Snyder‐Halpern & Verran, 1987), Pittsburgh Sleep Quality Index (Zhang et al., 2013), Sleep in Intensive Care Questionnaire (Freedman, Kotzer, & Schwab, 1999) and the Richards‐Campbell Sleep Questionnaire (RCSQ) (Richards et al., 2000) provide an indication of sleep. The advantages of measuring sleep quality using these questionnaires, bed‐side nurses and researchers could know patient's perception of night‐time sleep in a timely manner. However, these questionnaires with the exception of the RCSQ have some disadvantages: They have from 15–27 items. Furthermore, these instruments measure the quality of night‐time sleep before hospitalization. Therefore, it is not easy for critically ill patients to answer the items.

In particular, a simple, five‐item questionnaire, the RCSQ has been used in the United States and many countries. The RCSQ has been used as a surrogate indicator of sleep determined by PSG in the critically ill, but there is no established Japanese version of The RCSQ. This study aimed to determine the reliability and validity of the Japanese version of The RCSQ as a measure of sleep among ICU patients in a Japanese hospital.

## 2 BACKGROUND

The RCSQ shows a good correlation with PSG (Richards et al., 2000). The RCSQ has been translated into other languages including Spanish (Nicolas et al., 2008), Swedish (Frisk & Nordstrom, 2003) and German (Krotsetis, Richards, Behncke, & Kopke, 2017). Although the latter three versions have been verified for reliability, validity has not been fully evaluated. Evaluating sleep using the RCSQ is the most clinically relevant as the research in the lack for versatility of sleep evaluation using PSG. Therefore, the RCSQ is the most commonly used questionnaires in sleep research at ICU.

Although this questionnaire has previously been translated into Japanese (Tsuruta, Yamamoto, & Fujita, 2017), the reliability and validity or this Japanese translation have not been confirmed using standard guidelines on the translation process (Wild et al., 2005). Accurate measurement of sleep quality in critically ill patients using a validated Japanese version of the questionnaire will draw attention to developing a better understanding of sleep deprivation in the critical care environment. The Japanese version of the RCSQ (J‐RCSQ) also provides an instrument to evaluate interventions to improve sleep in the critically ill.

Our aim was to translate the English version of the RCSQ into Japanese using the back‐translation method and verify its reliability and validity in reference to a simplified measure of PSG. We hypothesized that the J‐RCSQ would be a reliable and valid measure of sleep quality in critically ill patients in Japan.

## 3 METHODS

### 3.1 Design

This study was cross‐sectional survey.

### 3.2 Setting

This study investigated the night‐time sleep of patients admitted to the general ICU in a university hospital between 22 August 2014–26 February 2015. The hospital had 1,075 beds in total, 20 beds in the semi‐closed ICU and 1:2 nurse‐to‐patient ratio.

### 3.3 Participants

Any patient aged 18 years or older who planned to stay one full night (9 p.m.–7 a.m.) in the ICU was eligible for inclusion. The exclusion criteria were as follows: positive delirium screening (using the Japanese version of the Intensive Care Delirium Screening Checklist: ICDSC ≧ 4) during the preceding night shift (Bergeron, Dubois, Dumont, Dial, & Skrobik, 2001; Koga, Murata, & Yamase, 2014), unstable hemodynamic and respiratory status requiring deep sedation, use of sleep medications during the investigation, mechanical ventilation, sepsis, shock, hepatic encephalopathy and renal failure.

Sample size calculations indicated that 29 patients were required to ensure 80% power, 5% significance and 0.50 effect size (the expected correlation coefficient) (Richards et al., 2000). The dropout rate of people in studies using PSG (including declined and wished to discontinue PSG) has been approximately 30% (Elliott, McKinley, Cistulli, & Fien, 2013; Richards et al., 2000). We anticipated a dropout rate of 30%–35% for this study. Therefore, we recruited 45 ICU patients who met the eligibility criteria. To determine sample size calculation, we used a program developed by the Clinical and Translational Research Program at the University of California, San Francisco.

### 3.4 Instrument development

#### 3.4.1 The RCSQ Instrument

The RCSQ was used to measure sleep quality for eligible ICU patients. The questionnaire includes a five‐item scale: (a) sleep depth; (b) time taken to fall asleep (sleep latency); (c) number of awakenings; (d) percentage of time spent awake; and (e) overall sleep quality. Each item is scored by using a 100‐mm visual analogue scale (0 mm: worst sleep, 100 mm: best sleep). Principal component analysis has confirmed that the RCSQ has a one‐dimensional structure (Richards et al., 2000). In other words, the total score on the RCSQ has been confirmed to represent the perception of sleep quality.

The validity and reliability of the RCSQ were confirmed by Richards et al. (2000), wherein 70 non‐ventilated ICU patients were included, and PSG was the evaluation criterion for sleep status. A Grass 16 Channel Electroencephalogram (EEG) Machine with 21 lead connections was used to objectively measure sleep. EEG, nasal and oral airflow, chest and abdominal excursion, electrocardiogram and anterior tibialis electromyogram (EMG) were measured. In the study, validity was determined by evaluating the association between sleep efficiency (SE) and total RCSQ score. The correlation coefficient between SE (measured using PSG) and the total RCSQ score was 0.58 (*p *< 0.001) (Richards et al., 2000). Further, for the English, Spanish, Swedish and German versions of the RCSQ, the Cronbach's coefficient alpha was 0.90 (Richards et al., 2000), 0.89 (Nicolas et al., 2008), 0.92 (Frisk & Nordstrom, 2003) and 0.88 (Krotsetis et al., 2017) respectively, thus, verifying the reliability of the questionnaire. Since no large difference in reliability was detected between the original and the three translated versions of the RCSQ, the questionnaire is considered to have high reliability.

#### 3.4.2 Developing the J‐RCSQ

After obtaining licensing from the author of the RCSQ, Dr. Kathy C. Richards, the J‐RCSQ was created according to the following procedure (Supporting Information Figures S1 and S2). We translated the RCSQ into Japanese with reference to the guidelines for the translation and adaptation of psychometric scales (Wild et al., 2005). The procedures included the following: (a) forward translation: Two translators carried out independent translations of the RCSQ from English into Japanese; (b) reconciliation: Three nursing science researchers had a discussion and reached a consensus on a draft of the J‐RCSQ that best reflected the literal and conceptual content of the original English version; (c) back translation: One native English‐speaking professional translator, with no knowledge of the original English version of the RCSQ, implemented back translation of the Japanese version into English; (d) back‐translation review: The primary investigator and another native English‐speaking professional translator, who was not involved in the translation process, reviewed the back translations against the original English version of the RCSQ; (e) harmonization: The primary investigator ensured the literal and conceptual equivalence of the translation; and (f) cognitive debriefing, review of cognitive debriefing results and finalization: Five consenting Japanese patients not participating in the study tested the J‐RCSQ and two authors reworded the phrases to improve comprehension.

### 3.5 Data Collection

#### 3.5.1 Data collected by PSG

We used a PSG (Alice PDx®; Philips Respironics) to accurately measure sleep stages. Three electrophysiological indicators for sleep measurements—EEG, electro‐oculogram (EOG) and submental EMG—were used (Berry et al., 2017; Murata & Yamaguchi, 2018).

The EEG electrodes were attached by the primary investigator who acquired the correct application method provided by a laboratory technician at the Philips's sleep centre. This technician was highly knowledgeable about the standard method of electrode application indicated by the international EEG society (10–20 electrode system). Nine post meridiem was defined as the time “to darken the environment in the ICU” (=light out), and “waking up” was defined as “when the people naturally woke up in the morning” or “to begin nursing care at 7 a.m.”(Murata & Yamaguchi, 2018). Time in bed (TIB) was defined as the time between the lights out and waking up.

Data were analysed by a laboratory technician of the Philips's sleep centre. The technician received masked patient information; sleep stage analysis was conducted with only the results of EEG, EOG and submental EMG.

The sleep stage analysis was in accordance with the classifications proposed by the American Academy of Sleep Medicine in 2012 (Berry et al., 2017; Murata & Yamaguchi, 2018). The duration of each sleep stage and wake after sleep onset (WASO) was calculated as the percentage of the sleep period time (SPT), defined as the amount of time in minutes from sleep onset until lights on in the morning, including all periods of waking in between. WASO was defined as the amount of waking after sleep onset until lights on in the morning (Murata & Yamaguchi, 2018). Total sleep time (TST) was defined as SPT excluding all periods of waking. Other variables calculated included TIB, sleep latency and SE. Sleep latency was defined as the period from lights off to the first three consecutive epochs of stage 1 sleep or an epoch of any other stage. SE was defined as the ratio of TST to TIB. Arousal index was defined as the number of arousals per hour during the TST.

#### 3.5.2 Data collection with the J‐RCSQ

Immediately after the completion of data collection with the equipment, the two investigators involved in the creation of the J‐RCSQ evaluated the people' sleep quality using the J‐RCSQ. People with impaired handwriting dictated their answers to the evaluator.

#### 3.5.3 Data collection from medical records

Data collected from medical records included age, gender, admission diagnosis, length of stay in the ICU and acute physiology and chronic health evaluation II score (APACHE‐II). In hospital that collected the data, APACHE‐II was used as an indicator of severity. Therefore, we decided to use APACHE‐II.

### 3.6 Statistical analysis

The total score of the J‐RCSQ was compared with the SE derived from the PSG data to determine the validity. To identify the correlation between the five items of the J‐RCSQ and the relevant sleep parameters (Richards et al., 2000) in the PSG, Pearson's correlation coefficient was used. The internal consistency (reliability) of the J‐RCSQ was tested using Cronbach's alpha coefficient. Demographic data were presented as mean (*SD*) or the number and its proportion. Translation of the questionnaire into Japanese may alter the association of each item in the PSG with the one in the RCSQ. Therefore, we evaluated the correlation of all five items as in the study of the original version (Richards et al., 2000). All statistical processing was performed with SPSS version 23 for Windows (IBM).

### 3.7 Research Ethics Committee approval

Approval was received from the institutional review board of The Jikei University in Japan to collect data at the affiliated hospital (reference number: 26‐024(7529)). Informed consent was obtained from all participants prior to their inclusion in the study.

## 4 RESULTS

Of the 45 eligible participants, five declined participation and seven were excluded because their electrodes came off during the study. The participants consisted of 23 men and 10 women aged 69.1 (*SD*: 8.8) years. The average score for the APACHE‐II was 12.4 (*SD*: 4.3; Table 1). Cronbach's alpha coefficient for the J‐RCSQ was 0.911.

**Table 1:** Characteristics of the 33 patients at enrolment

Gender, *N* (%)	
Male/Female	23 (69%)/10 (31%)
Age, mean ± *SD*	69.1 ± 8.8
APACHE‐II, mean ± *SD*	12.4 ± 4.3
Intensive care unit stay days until investigation, mean ± *SD*	1.2 ± 0.6
Admitting reason	
Surgical (elective)/Medical	28 (85%)/5 (15%)
Admission diagnosis category, *N* (%)	
Lung cancer	11 (33.3)
Cerebral vascular disease	8 (24.2)
Cardiovascular	4 (12.1)
Knee osteoarthritis	4 (12.1)
Respiratory failure	3 (9.1)
Digestive organ cancer	2 (6.1)
Acute nephritis	1 (3.0)

Note

APACHE‐II: acute physiology and chronic health evaluation II, *SD*: standard deviation.

No participant met the criteria for delirium (at least four points) in a night‐time delirium assessment using the Japanese version of the ICDSC (Koga et al., 2014). However, four people were considered to have subsyndromal delirium (subsyndromal delirium: ICDSC = 3 points). Table 2 shows the means of the visual analogue five items of the J‐RCSQ and that of the corresponding parameters derived using PSG for all the 33 participants (Table 2A) and 29 participants with ICDSC < 3 points (Table 2B). Correlations between the five J‐RCSQ items and the PSG sleep characteristics are also presented in Table 2A,B.

**Table 2:** (A) Descriptive statistics and correlation of PSG sleep characteristics with the RCSQ items. (B) Descriptive statistics and correlation of PSG sleep characteristics with the RCSQ items (The group that excluded the four people with subsyndromal delirium)

Sleep domains	J‐RCSQ item	J‐RCSQ item mean (*SD*)	PSG sleep characteristics	PSG mean (*SD*)	*r* a
(A)					
Sleep depth	Q.1 Sleep depth	41.01 (21.67)	REM (%)	2.63 (4.38)	−0.005
			N1 (%)	66.95 (27.55)	−0.269
			N2 (%)	30.32 (26.81)	0.275
			N3 (%)	0.082 (0.360)	0.137
Falling asleep	Q.2 Sleep latency	43.09 (27.29)	Sleep latency (min)	35.52 (51.41)	−0.408*
Number of awakenings	Q.3 Awakening	28.86 (19.96)	Arousal Index (times/hr)	49.95 (25.24)	−0.255
			TST (min)	296.83 (142.03)	0.266
Percent of time awake	Q.4 Returning to sleep	45.41 (25.92)	Ratio of WASO (WASO/SPT; %)	46.64 (22.97)	−0.423*
			TST (min)	296.83 (142.03)	0.471*
Quality of sleep	Q.5 Sleep quality	40.58 (22.27)	Sleep latency (min)	35.52 (51.41)	−0.318
			TST (min)	296.83 (142.03)	0.265
			Arousal Index (times/hr)	49.95 (25.24)	−0.164
			Ratio of WASO (WASO/SPT; %)	46.64 (22.97)	−0.238
			REM (%)	2.63 (4.38)	−0.121
			N1 (%)	66.95 (27.55)	−0.118
			N2 (%)	30.32 (26.81)	0.140
			N3 (%)	0.082 (0.360)	0.028
Overall	J‐RCSQ Total	39.79 (20.25)	Sleep efficiency (%; TST/TIB)	49.44 (23.87)	0.459*
(B)					
Sleep depth	Q.1 Sleep depth	43.16 (21.57)	REM (%)	2.40 (4.34)	−0.026
			N1 (%)	69.37 (27.68)	−0.325
			N2 (%)	28.13 (27.38)	0.330
			N3 (%)	0.072 (0.371)	0.140
Falling asleep	Q.2 Sleep latency	46.43 (26.91)	Sleep latency (min)	39.43 (53.60)	−0.540*
Number of awakenings	Q.3 Awakening	30.28 (20.46)	Arousal Index (times/hr)	51.14 (26.47)	−0.275
			TST (min)	284.12 (144.32)	0.329
Percent of time awake	Q.4 Returning to sleep	48.00 (25.61)	Ratio of WASO (WASO/SPT; %)	48.78 (23.55)	−0.526*
			TST (min)	284.12 (144.32)	0.572*
Quality of sleep	Q.5 Sleep quality	44.11 (20.69)	Sleep latency (min)	39.43 (53.60)	−0.494*
			TST (min)	284.12 (144.32)	0.408*
			Arousal Index (times/hr)	51.14 (26.47)	−0.219
			Ratio of WASO (WASO/SPT; %)	48.78 (23.55)	−0.388*
			REM (%)	2.40 (4.34)	−0.175
			N1 (%)	69.37 (27.68)	−0.204
			N2 (%)	28.13 (27.38)	0.233
			N3 (%)	0.072 (0.371)	0.033
Overall	J‐RCSQ Total	42.39 (19.51)	Sleep efficiency (%; TST/TIB)	47.38 (24.37)	0.602*

Notes

Arousal index: defined the number of arousals per hour during the TST; J‐RCSQ: Japanese version of the RCSQ; N1 (%): time of non‐rapid eye movement sleep (NREM) stage 1/TST; N2 (%): time of NREM stage 2/TST; N3 (%): time of NREM stage 3/TST; PSG: polysomnography; Q: question; RCSQ: the Richards‐Campbell Sleep Questionnaire; REM (%): time of rapid eye movement sleep/TST; *SD*: standard deviation; sleep efficiency: the ratio of TST to TIB; sleep latency: time taken to falling asleep; SPT: sleep period time; TIB: time in bed; TST: total sleep time; WASO: wake after sleep onset.

^a^Correlation between the EEG sleep characteristics and corresponding RCSQ Item. *p < 0.05.

**Figure 1:**
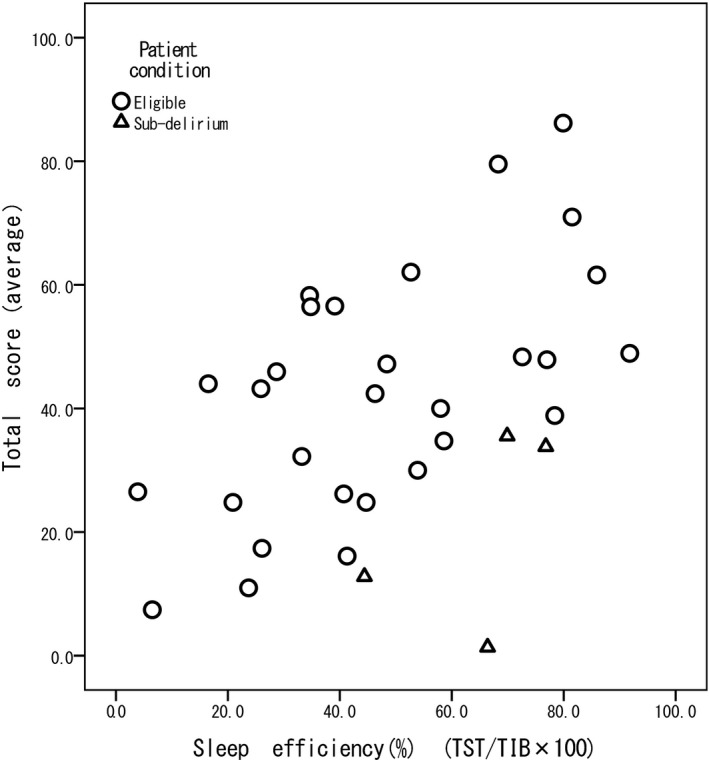
Correlation between sleep efficiency and the total score on the Japanese version of the Richards‐Campbell Sleep Questionnaire

The group excluding four patients with subsyndromal delirium had a higher correlation coefficient than the group including them (Table 2). No correlation was observed for any items in sleep depth (Q1) or awakening (Q3). For sleep latency (Q2), a moderate correlation was observed with sleep latency (*r* = −0.408 or −0.540, *p *< 0.05). For returning to sleep (Q4), a moderate correlation was observed with the ratio of WASO (=WASO/SPT, *r* = −0.423 or −0.526, *p *< 0.05) and TST (*r* = 0.471 or 0.572, *p *< 0.05). For sleep quality (Q5), in 29 patients not having subsyndromal delirium, correlations with sleep latency, ratio of WASO and TST were observed (*r* = −0.494, *p *< 0.05; *r* = −0.388, *p *< 0.05; *r* = 0.408, *p *< 0.05, respectively). The relationship between the total J‐RCSQ score and SE is shown in the scatter plot (Figure 1). The Pearson correlation coefficient between SE and the total J‐RCSQ score was 0.459 (*p *= 0.007). After excluding the four patients with subsyndromal delirium, the Pearson correlation coefficient was 0.602 (*p* = 0.001).

## 5 DISCUSSION

The reliability of the J‐RCSQ measured using Cronbach's alpha coefficient was 0.911 and 0.90 for the original English version (Richards et al., 2000). The validity of J‐RCSQ was confirmed by the moderate correlation between SE as measured by the simplified PSG and the total J‐RCSQ score (correlation coefficient: 0.459–0.602). The correlation for the original RCSQ and PSG was 0.58. These results suggest that the J‐RCSQ is as valid and reliable for the evaluation of sleep in non‐intubated, non‐delirious Japanese patients admitted in the ICU, as the original version for American patients. The three translated versions of the RCSQ (the Spanish, Swedish and German versions) have been verified for the reliability by previous studies (Frisk & Nordstrom, 2003; Krotsetis et al., 2017; Nicolas et al., 2008), while their validities have not been evaluated with reference to PSG. In the current study, in addition to the reliability, the validity of the Japanese version was evaluated using simplified PSG.

Compared with previous research, sleep latency (Q2), one of the PSG sleep characteristics, was more highly correlated with the J‐RCSQ than the original English version (*r* = −0.17) (Richards et al., 2000). This difference could be due to the environment and participants. Although both studies were performed in an ICU, the study by Richards et al was performed in a medical ICU including male veterans with no information on patient severity scores, while the current study included both male and female participants, most of whom were surgical with relatively low APACHE‐II scores. In the current study, however, the correlation between the APACHE‐II score and the RCSQ was not evaluated since the sample size did not allow for a valid multivariate analysis.

Further, there was no correlation between the J‐RCSQ scores (Q1 and Q5) and the amount of restorative sleep in PSG including REM sleep and stage N3. These results may be due to a marked decrease in REM and N3 sleep of ICU patients (Freedman, Gazendam, Levan, Pack, & Schwab, 2001). The average Percent of REM was 2.6% or 2.4% and that of N3 sleep was 0.082% or 0.072% of the TST, respectively (Table 2).

In addition, the correlation coefficient was higher in the group that excluded the four people with subsyndromal delirium compared with all the participants. This could be because the four people with subsyndromal delirium might have responded to the questions without fully understanding them, since their night‐time lucidity was low. In other words, subjective evaluation of sleep quality by these subclinical delirious patients may not have been reliable, which could be a potential confounding factor when evaluating the relationship between sleep disturbance and delirium. In fact, a recent study evaluating sleep quality with questionnaires failed to show an association between sleep disturbance and delirium (Kamdar et al., 2015).

### 5.1 Limitations

Several limitations need to be addressed for the current study. First, variability existed in patient backgrounds, previously prescribed medications and ongoing disorders in a small patient group. Although efforts were made to exclude patients with these potential confounding factors, the results of the current study might underestimate or overestimate the correlation between the RCSQ and PSG results. Second in the present study, as shown in APACHE‐II scores, people were less severely affected than common ICU patients (e.g., intubated patients were excluded). Therefore, generalizability of the results to critically ill patients (e.g., patients with multiorgan system failure) was limited. To verify the validity of the J‐RCSQ in a wide range of patients is a challenge for future research.

## 6 CONCLUSIONS

As a sleep assessment tool in lucid patients in the ICU, the reliability and validity of the J‐RCSQ are similar to the original RCSQ. Consequently, the J‐RCSQ can be used as an alternative to PSG when assessing sleep quality in non‐ventilated postoperative ICU patients.

## CONFLICT OF INTEREST

The authors declare no conflict of interest.

## AUTHOR CONTRIBUTIONS

HM: Study plan, data collection and overall manuscript writing. YO, KS, YY and MT: Conception and design of the work. Additionally, MS, KR and RH: Interpreting data and revising this manuscript critically. All authors meet the criteria for authorship and have approved the final article.
