# Supplementary material for: The Japanese version of the Richards‐Campbell Sleep Questionnaire: Reliability and validity assessment
Source: Nurs Open. 2019 Mar 28;6(3):808–14. doi: 10.1002/nop2.252 (PMC6650758; doi:10.1002/nop2.252)
Supplement: Supplementary file 1 [file NOP2-6-808-s001.docx]

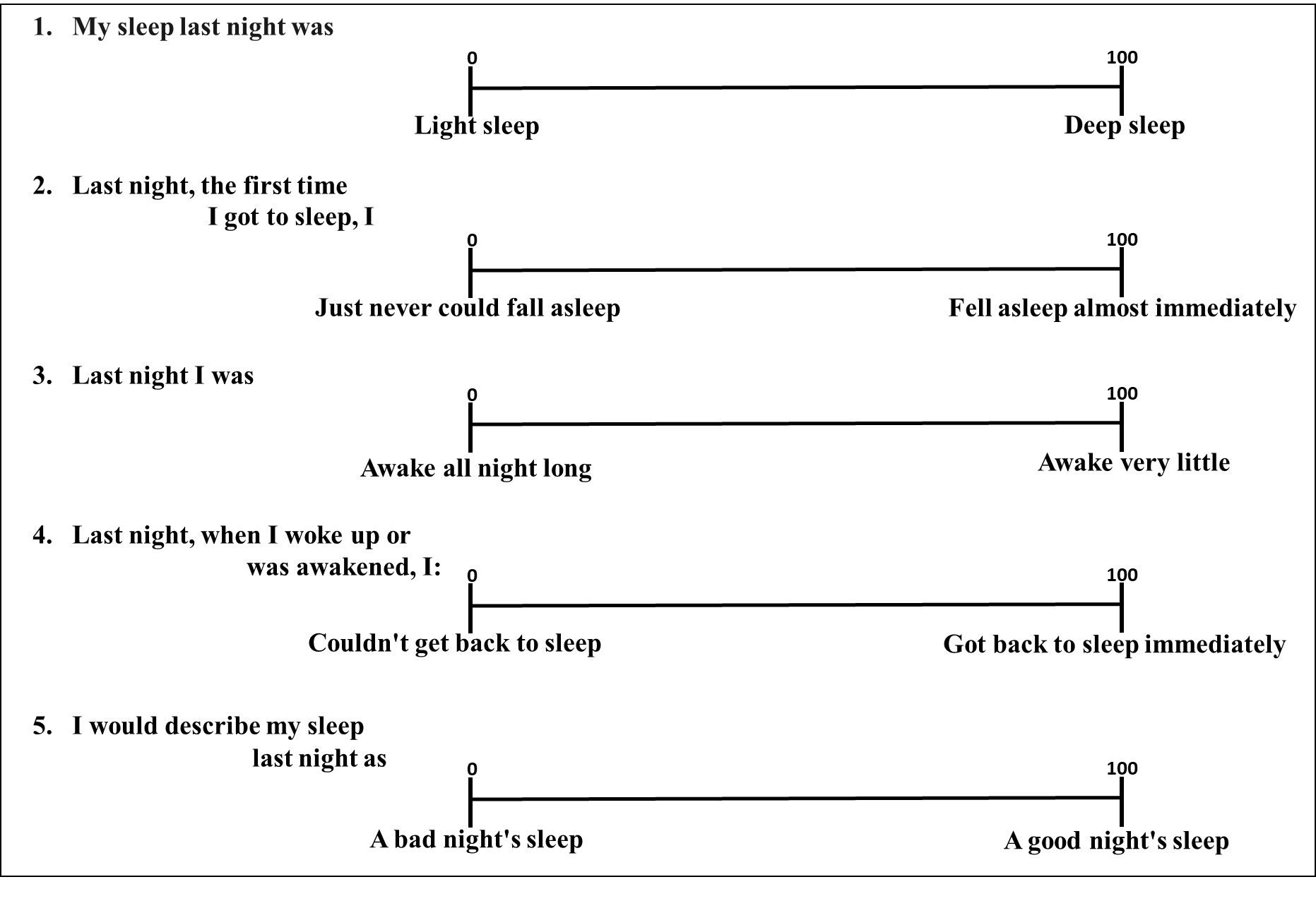


**Figure S1. Original English version of the Richards-Campbell Sleep Questionnaire**


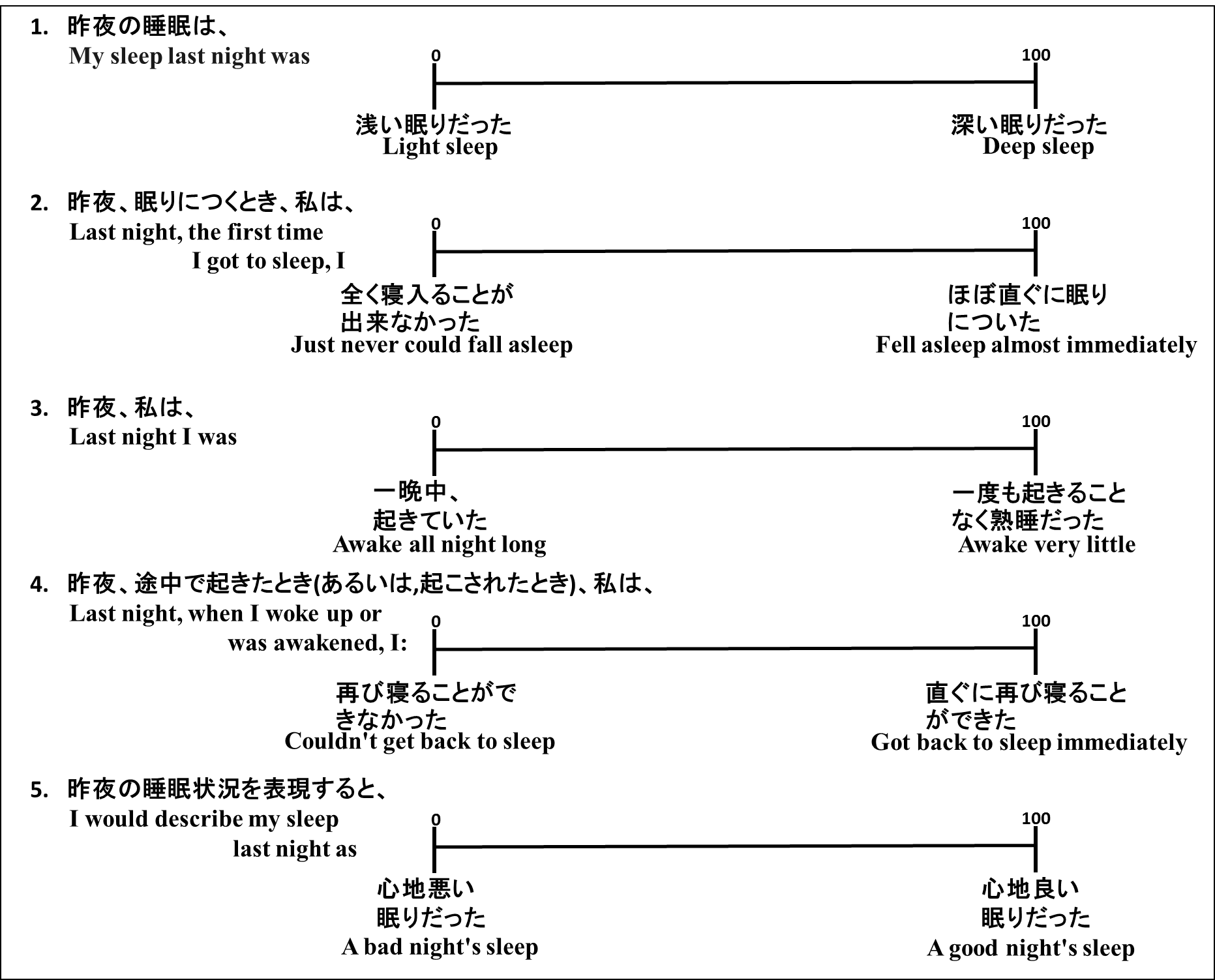


©<<School of Nursing at Narita, International University of Health and Welfare, Chiba, Japan / Hiroaki Murata>>: reproduced/translated with kind permission of << School of Nursing, University of Texas at Austin / Kathy C. Richards>>

**Figure S2. The Japanese version with English translation of the Richards-Campbell Sleep Questionnaire**
